# OTP-PRL: an app for occupational risk prevention in policing activities

**DOI:** 10.1186/s12889-019-7935-2

**Published:** 2019-11-21

**Authors:** José C. Vera-Jiménez, Marta Ferreiro-González, Gerardo F. Barbero, José Ángel Álvarez, Francisco Fernández-Zacarías, Jesús Ayuso

**Affiliations:** 1 Cadiz Municipal Police, Police Technology Area, Public Safety School, Cadiz Council, Cadiz, Spain; 2 Department of Analytical Chemistry, Faculty of Sciences, IVAGRO, CeiA3, University of Cadiz, P.O. Box 40, 11510 Cadiz, Puerto Real Spain; 3 Department of Physical Chemistry, Faculty of Sciences, Institute of Biomolecules (INBIO), University of Cadiz, 11510 Cadiz, Puerto Real Spain; 4 Department of Industrial and Civil Engineering, University of Cadiz, 11510 Cadiz, Puerto Real Spain

**Keywords:** Smartphone application, Information and communication technologies, Occupational risk prevention, Police profession, Android, iOS

## Abstract

**Background:**

The rapid progress in information and communication technologies has promoted the development of smartphone applications (apps) for a wide variety of purposes including workplace safety. However, no specific apps for occupational risk prevention in the police domain seemingly exist as yet. In this work, an app running under the iOS and Android operating systems was developed to help police officers become acquainted with policing-related occupational risks and to prevent their damaging consequences.

**Results:**

The proposed app, which uses an iterative user-centered design to avoid occupational risks in policing activities, was assessed for performance by a group of users and experts through a System Usability Scale (SUS) questionnaire. The mean overall score for the questionnaire was 82.3. The app has backend support to facilitate continual improvement through contributions from users and administrators. A field test revealed increased awareness of policing occupational risks after using the app in many users.

**Conclusions:**

A novel product that covers the needs of ORP requirements of police officers has been developed.

## Background

The widespread implementation of information and communications technologies (ICTs) has promoted the development of a myriad of smartphone applications (apps) for a wide variety of purposes. Currently available smartphone apps span a vast but still growing range of activities. Smartphones are now equipped with powerful operating systems such as iOS or Android that allow users to access additional software, processing capabilities and a number of network connectivity options [1]. One or more apps currently exist for virtually every imaginable purpose from basic gaming to serious business, financial management or even preventive healthcare and safety. For example, there are apps for measuring noise level [2], calculate thermal comfort [3] and estimate stress or anxiety [4]. Some studies have shown that mobile health apps can be very useful to assure workplace health [5]. The growing use of health-related apps is changing the traditional face-to-face way of interacting. Digitization, connectivity and interconnection of machines, products, services or even workers have all had a strong impact on many fields.

The number of health-related apps for the two major operating systems (iOS and Android) exceeded 100,000 in 2014 [6] and has continued to rise. In fact, cell phones have created an opportunity to promote healthy behaviors worldwide. For example, the fact that noise has considerable social and physiological impacts has led some authors to assess the usability of smartphones for raising awareness of its associated hazards [6–9]. Some apps can accurately measure noise with intensity differences lower than 0.52 dB as compared to a calibrated sound level meter (SLM) [2, 8, 10] and can thus be very useful to assess hearing-related occupational risks.

Properly managing risks is essential with a view to anticipating potential threats and to providing the tools needed to minimize their impact. A safe, healthy working environment is an essential condition for assuring quality of life [11].

Although trade unions and public administrations have long strived to increase workplace safety by promoting occupational risk prevention (ORP), work accidents continue to occur and to be the subject of much research across the world [12, 13]. Thus, in 2017 there were 596 work accidents in Spain only [14]. Workplace safety is not only a humanitarian concern, but also an economic one [12]. For this reason, improving working conditions remains a global challenge and one of the main goals of the European Union (EU) as set in Article 153 of its treaty [15].

Police officers have a high-risk job relative to many other workers. Policing risks vary depending on the particular duty. Because emergency situations invariably involve some hazard or even extreme danger, policing is a relatively complex target for risk prevention [12, 16–18]. The most serious risks of policing activities examined to date include musculoskeletal disorders [19], attacks during assaults and homicides in acts of service [16, 20], traffic accidents and work-related stress [21, 22], depression and even suicide [23]. In fact, police officers worldwide are subject to a number of psychosocial occupational risks [24] in addition to fatigue and stress derived from chronic auditory, respiratory or inflammatory diseases [25–28], or dermatological problems [29] resulting from certain habits, operational procedures or postural behaviors. Most such risks can be avoided, however.

Governments and the scientific community are striving to prevent policing risks. Progress in the police domain, however, has been slow relative to other professions. The most salient studies on policing-related ORP have examined the effectiveness of a nap of 30–90 min before a night shift to combat fatigue and drowsiness [30]; that of orthoses in preventing lower limb injuries [31]; the effects of risk prevention regulations [32]; the influence of food, stress and daily exercise, among other factors, on health [33]; the efficacy of reducing alcohol consumption and increasing fruit intake in addition to other lifestyle changes in reducing hypertension in officers [34]; the role of work-related emotions in the relationship between fatigue and exhaustion [35]; the strong correlation between physiological variables and stress measures [7]; and the effectiveness of UV protection [29].

A literature search for iOS and Android apps for ORP retrieved a large number of references for some professions but none for policing. This lack of apps for police activities led us to develop a dedicated multifaceted ORP app for this domain.

Google Play Store offers a number of occupational health and safety (OHS) apps including five developed by the Spanish National Institute of Occupational Safety and Health, but, again, none for policing-related risks. Such apps run under three different operating systems (iOS, Android and Windows) and are completely free [36]. The primary goal of this work was to develop an app running under iOS and Android that would (*a*) provide preventionists with a tool for assessing potential occupational risks of specific policing tasks; (*b*) advise police officers of the risks they may encounter in order to raise their awareness of policing-related occupational risks and train them to prevent their damaging consequences (all the contents of the app are intended to be used as several tools of formation and clarification of doubts and problems, etc., only when the police are not involved in active operations).The app was subjected to consultations with police officers to receive their complaints and suggestions in order to increase the effectiveness for accomplishing health-related behavior changes in their performance.

Typical specific policing tasks include Special Units, Public Safety, Traffic Officer, Neighborhood Policing, and Attestations.

## Methodology

The methodology and procedures followed for the implementation of the present app for occupational risk prevention in policing activities can be distinguished into three stages:

- App design
- Version analysis and design
- Pilot study

### App design

Policing risks depend on the particular task to be performed. The most likely risks arise from physical activities and include accidents and the consequences of poor movement technique, attacks, aggression, overexertion, fatigue, dissatisfaction and burnout.

**Fig. 1:**
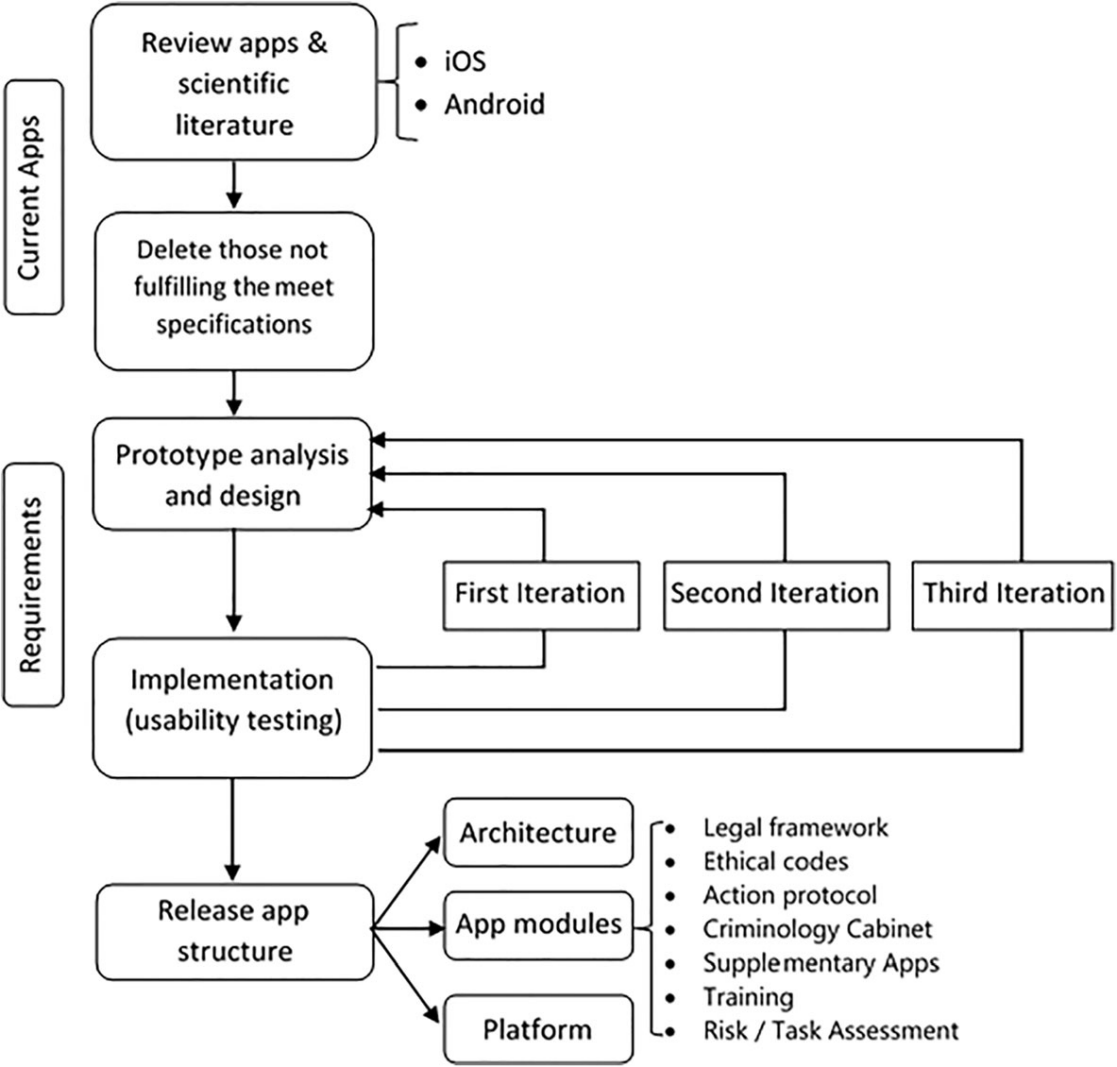
Methodology used to design the proposed app

A dedicated, comprehensive app for addressing the most common serious risks of policing was developed in several steps (see Fig. 1). The first step involved examining existing apps for similar purposes in other occupational fields and conducting an extensive literature search to identify ways of improving some capabilities as regards preventing policing risks and to exclude any apps not meeting the specifications. The initially selected apps were classified according to operating system (iOS or Android) and examined for design and functioning.

The large variety of designs spanned by the apps shared a common development sequence in accordance with standard guidance [37], namely: conceptualization (formalization of the idea, research), design (wireframes, prototypes, user tests, visual appearance), development (code programming, debugging) and release (launching, follow-up, updates). Each type of design was found to have its own advantages depending on the particular aim and on the developer’s experience and programming style. The app most closely fitting the intended purpose was one revolving around a qualitative, user-centered approach [38]. As advised by a group of experts, a flexible model was used to ensure that the proposed app would run equally well under iOS and Android.

Because the app was aimed at improving occupational risk prevention among law enforcement agents, and should conform to existing legislation and be applicable in all areas of policing (e.g., driving, exposure to toxic environments, special interventions), the authors contacted an expert group for advice on designing a rough version. The expert group comprised three OR preventionists, two criminologists versed in applicable policing OR legislation, one mechanical engineer, one computer engineer, two chemists and three police officers with experience in risk prevention. The initial meeting of the group was followed by several working sessions to check whether the app met the target specifications as regards the variety of policing tasks to be addressed and prevention of their associated risks. The app was designed mainly to deal with police risks outside the office as it is during patrolling that officers are exposed to the greatest risks —and largely unprotected as regards prevention.

### Version analysis and design

**Fig. 2:**
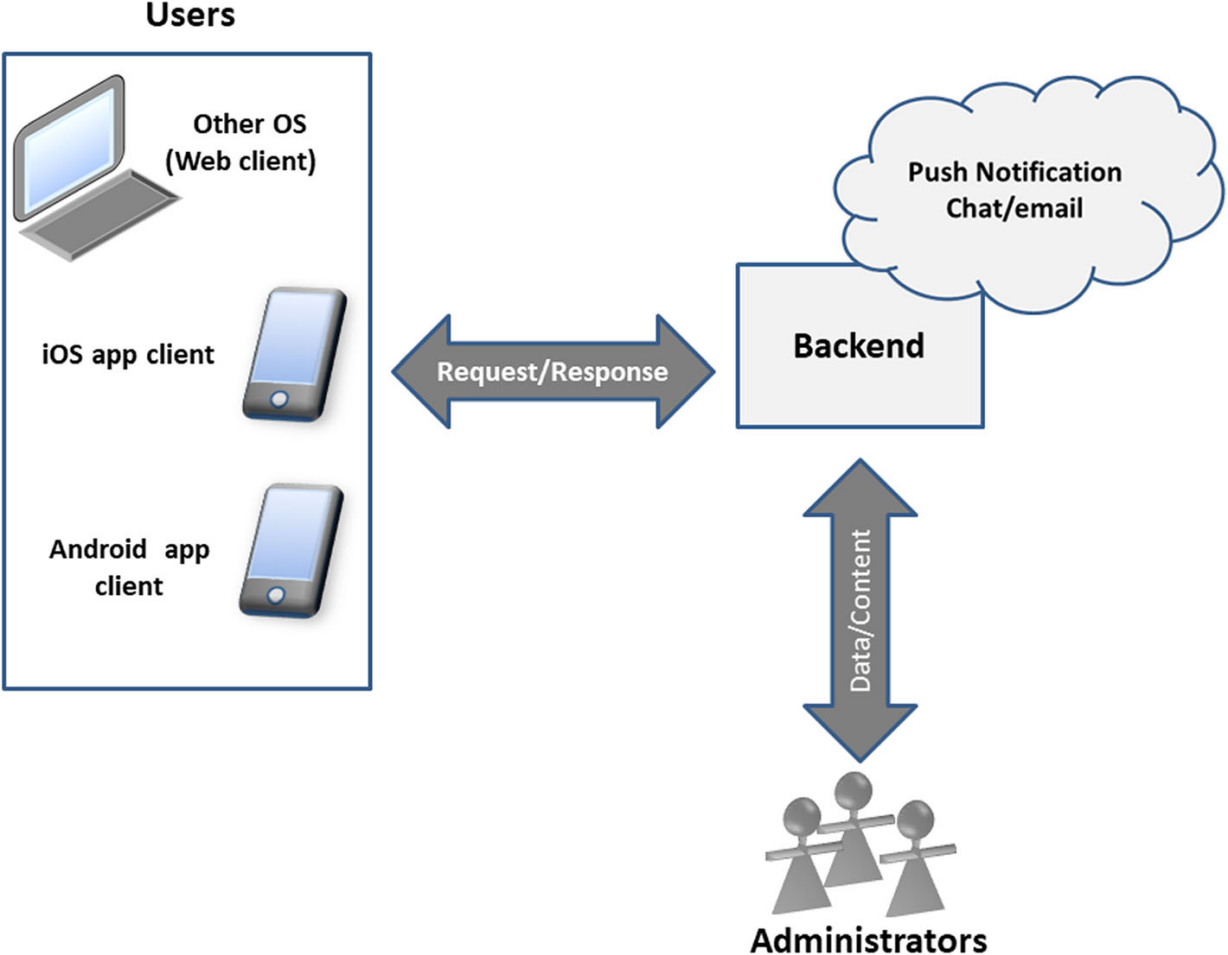
System architecture of the app

A version analysis was performed and a preliminary design developed to ensure accessibility, agile content maintenance and compatibility of the app with most smartphones. Computer engineers and graphic designers captured all initial ideas in a draft version of the app for iOS and Android. The final system architecture comprised a user app, a content management platform (backend) and an additional app for advisors and administrators (see Fig. 2). Using backend support was intended to facilitate future improvement of the app with contributions from users and administrators. There are no differences between using iOS and Android.

**Fig. 3:**
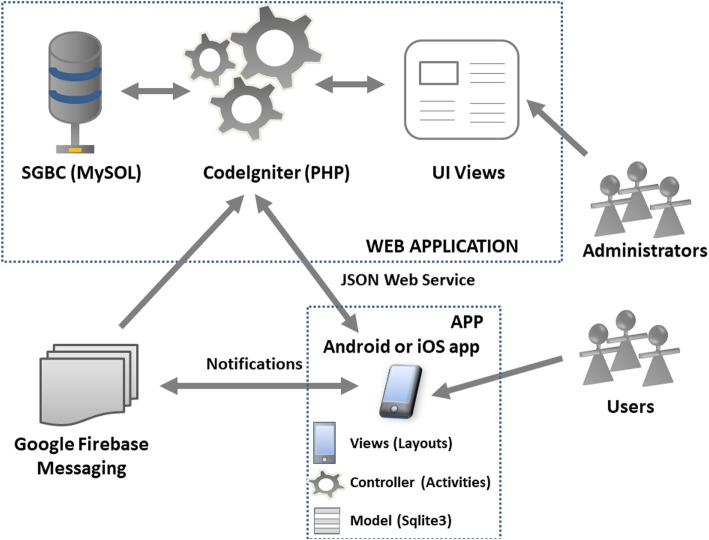
Content management system of the app

The platform management system used allows the whole app content to be controlled, but especially content creation and maintenance, and management of app instances. The platform uses PHP technology [39, 40], which is free and open-source; also, its developer environment can be easily set up, and databases rapidly accessed. The specific software used, MySQL, is operationally expeditious and uses modest resources, so it can be run on low-end devices (see Fig. 3). The app was developed in JAVA language for iOS and Android. The most recent content version retrieved from the administrator was stored by using SQLite, which affords off-line running of apps, on the smartphone end.

### Pilot study

In 1986 John Brooke at Digital Equipment Co. Ltd., developed a questionnaire, the System Usability Scale (SUS) that could be used to take a quick measurement of how people perceived the usability of computer systems on which they were working [41]. This proved to be an extremely simple and reliable tool for when doing usability evaluations, and John Brooke decided that it was probably something that could be used by other organizations, making SUS freely available to who might find it useful.

#### Usability study

The app was evaluated for usability by three groups of 12, 15 and 17 officers of the municipal police of Cadiz, Spain. The purpose was to check whether the app version met the users’ functional requirements and expectations, and also to identify potentially improvable function and design features. Usability was evaluated by using various participatory and user-centered design techniques including focus groups, and usability and heuristic evaluation methods.

Group sessions were used to describe the initial version of the app and to instruct users in its handling. Subsequent individual sessions were used to monitor app usage. Users were encouraged to comment on any aspects of the app or its handling they thought fit.

Each individual session was followed by interviews with the users and completion of a System Usability Scale (SUS) questionnaire based on a 5-point Likert scale [41]. This scale was presented to the users with a series of statements and they expressed their degree of agreement or disagreement. All participants that took part in the SUS questionnaire were informed that the test was anonymous and there was no question related to them. The SUS questionnaire only incorporate questions on the ease of use and management of the APP but without omitting any statement or judgment. Verbal consent from the participants was obtained in accordance with the Organic Law 3/2018, of December 5, on the Protection of Personal Data and guarantee of digital rights [42].

Usability scales have been used to evaluate a wide variety of products and services including hardware and software. They are very easy to administer to participants; also, they can be reliably used with small sample sizes, and allow usable and unusable systems to be discriminated [41] with greater accuracy than with alternative tests such as QUIS, CSUQ, Words or Ours. Table 1 lists the ten questions used to evaluate the app, the average score and standard deviation for each question in the 3 cycles. The versions were refined iteratively over 3 cycles, each cycle leading to a new and improved version.

**Table 1:** SUS scores for each iterative cycle in the tests

SUS questions	Scores cycle 1	Scores cycle 2	Scores cycle 3	*F* ratio	*p*
Q1. Would use the app frequently	3.5/0.5	3.8/0.6	3.8/0.4	2.5	NS
Q2. App unnecessarily complex	1.8/0.5	1.8/0.5	1.5/0.5	1.8	NS
Q3. App easy to use	3.6/0.5	4.2/0.7	4.3/0.5	7.9	.001
Q4. Need support to use the app	2.1/0.6	1.8/0.8	1.8/0.6	0.9	NS
Q5. Functions in app well integrated	3.4/0.6	3.8/0.6	4.3/0.5	12.6	<.001
Q6. Too much inconsistency in the app	2.4/0.6	2.2/0.7	1.7/0.5	7.7	.001
Q7. Most people would learn to use this app quickly	3.9/0.7	4.2/0.8	4.4/0.5	2.6	NS
Q8. Found the app very cumbersome to use	2.3/0.6	1.9/1.0	1.2/0.4	10.1	<.001
Q9. Felt very confident using the app	3.4/0.6	3.9/0.9	4.2/0.4	7.8	.001
Q10. Need to learn a lot before going with the app	2.7/0.5	2.3/1.0	2.1/0.7	3.4	<.05
*Global Score*	65.9/5.0	74.3/10.0	82.1/3.3	24.7	<.001

*NS* Not significant

To calculate the SUS-Score (SUS.S), called as *Global Score* in Table 1, first the sum of the score contributions from each item was performed. Each item’s score contribution was range from 0 to 4. For items 1,3,5,7, and 9 the score contribution was the scale position minus 1. For items 2,4,6,8 and 10, the contribution was 5 minus the scale position. By multiplying the sum of the scores by 2.5 the overall value of SU was obtained. The SUS.S (as *Global Score*) values ranged from 0 to 100.

Effectiveness was assessed in terms of the task completion rate, and of the frequency of incidents and data entry errors. After a one-month trial period, the shortcomings noted by the experts were referred to the computer engineers to improve the app.

An elementary test analysis of variance (ANOVA) was also performed to check whether differences in distribution were significant at *p value* = 0.05 in terms of the SUS.S as study variable.

## Results

### Advantages of the app

**Fig. 4:**
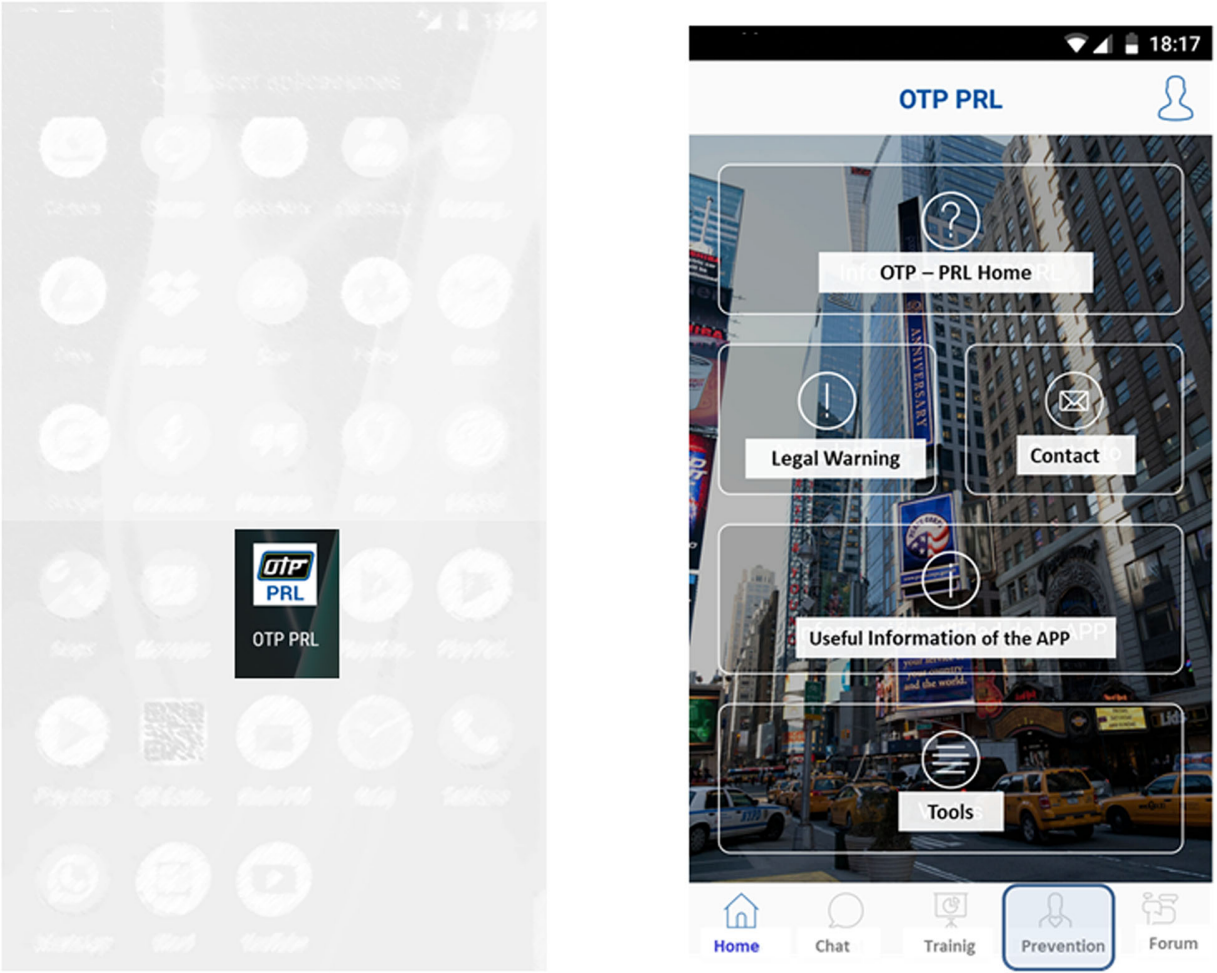
Screenshots of the app: OTP-PRL Icon (left); Home menu (right)

As can be seen in the screenshot of its Home menu (Fig. 4), the proposed app comprises various modules. Although we focused on risk assessment in the police domain, each module is briefly described below.

- *OTP-PRL Home* describes the background and scope of the app.
- *Legal Warning* lists all rights and legal matters.
- *Contact* provides users with an email address to contact the Administrator.
- *Useful information of the App* provides precise information about the uses and potential of the app.
- *Tools* comprises three different sub-modules, namely:
  1. A legal and informative module supplying general and policing-specific information on ORP legislation and regulations, as well as police ethical codes and intervention protocols.
  2. A criminological advice module that allows police officers to request criminological, legal medicine and forensic advice, as well as information on occupational risks, training and use of force, all by chat.
  3. Supplementary third-party apps for assessing specific risks, both general and specific to tasks such as storage of hazardous chemicals.

The main menu includes shortcuts for major tasks such as Chat, Training, Prevention and Forum.

- A *training module* for semi-distance or distance e-learning intended to help resolve conflicts arising during policing.
- A *chat module* for direct contact with an expert.
- A *forum for group discussion module* that is open to all users.
- A *risk assessment module* that is accessible by experts (preventionists) but not by regular officers and is described in detail below since it is the main innovation of the app.

### Risk assessment module

**Fig. 5:**
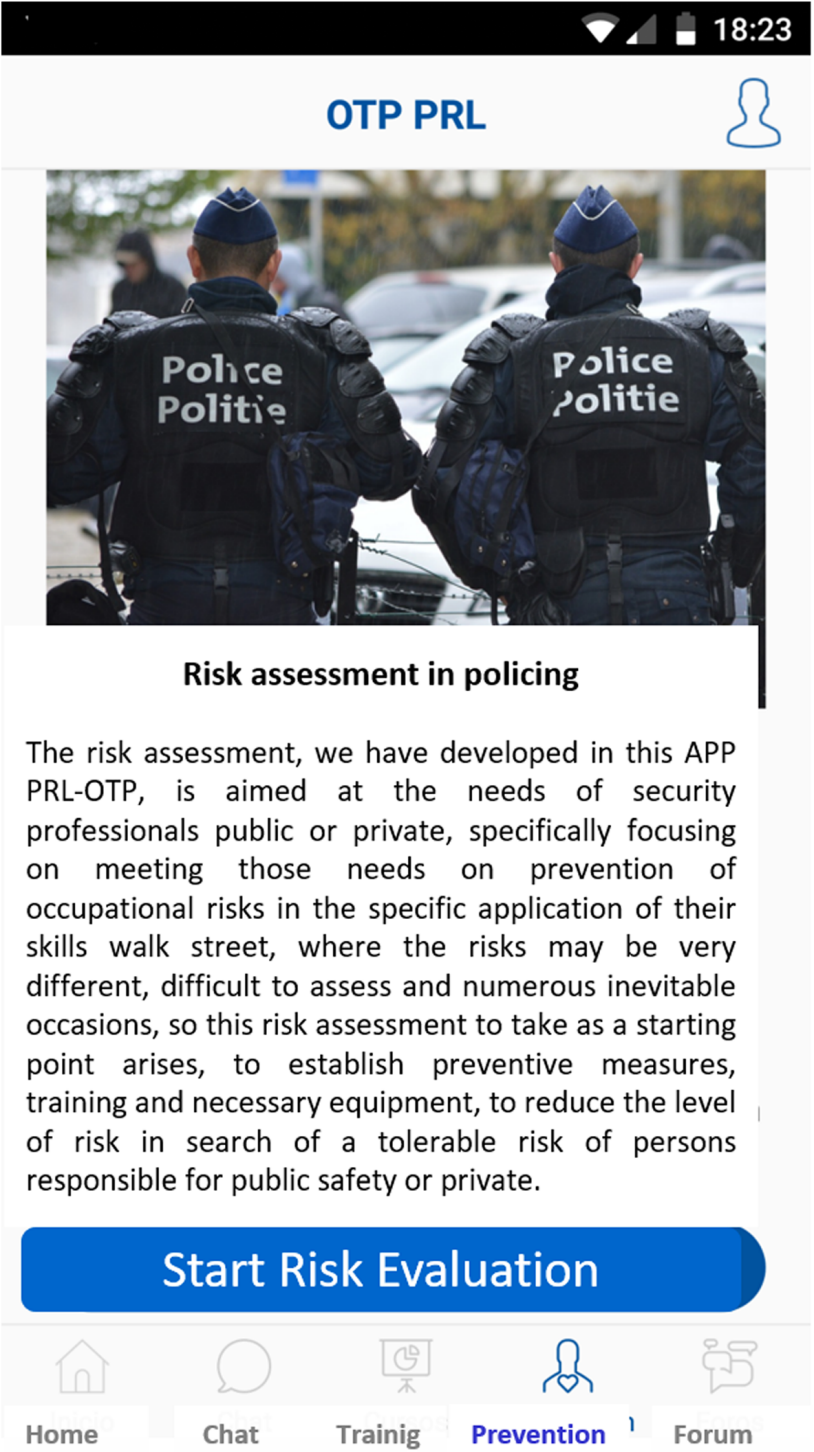
Screenshot of the OTP-PRL risk assessment module

The risk assessment module (Fig. 5) provides a guided system for recording occupational risks associated with a given hazard and for reporting assessment outcomes by e-mail. However, law enforcement officers will only access the expert consulting chat when necessary, not on police demand. As noted in the Introduction section, the occupational health risks faced by police officers depend on the particular policing task. Because risks can be physical or psychological, and range from mild to fatal in severity, the main purpose of this module is to assess the risks and dangers associated to each police action with provision for a large number of variables and factors in each case.

The experts insisted that this module should be easily navigated via drop-down menus to locate the risks or hazards associated to specific policing tasks. The app includes the following information options:

1. *Enforcement agencies:* Municipal Police, State Police, Civil Guard, Regional Police, Customs Agency, Port Police and Private Security.
2. *Calendar*.
3. *Job:* special units, public safety, traffic officer, neighborhood policing, attestations, others.
4. *Work shift:* morning, afternoon, night or rotating (morning– afternoon, morning–afternoon–night).
5. *Task*, which includes the following information:
  Tasks performed, duration and frequency.
  Places where work is conducted.
  The person performing each task, whether regularly or occasionally.
  Other individuals who might be affected by police work activities (visitors, subcontractors, and the public).
  Any training officers have received to perform the tasks.
  Written work procedures and/or work permits.
  Facilities, machinery and equipment used.
  Hand-operated power tools used.
  Manufacturer and supplier instructions for operating and maintaining machinery and equipment.
  Size, shape and nature of the work surface, and weight of the materials to be handled.
  Distance and height to which materials are to be moved by hand.
  Sources of energy used (e.g., compressed air).
  Substances and products used and produced at work.
  Physical state of the substances (fumes, gases, vapors, liquids, dust, solids).
  Label content and recommendations for the substances.
  Existing legal requirements as regards work conduct, facilities, machinery and substances.
  Control measures in force.
  Action in preventing occupational risks (incidents, accidents, activity-derived occupational diseases, equipment and substances used) as obtained from internal and external sources.
  Data on existing risk assessments for each activity.
6. *Organization of work*.
7. *Number of workers*.
8. *Type of evaluation:* initial or periodic.

**Fig. 6:**
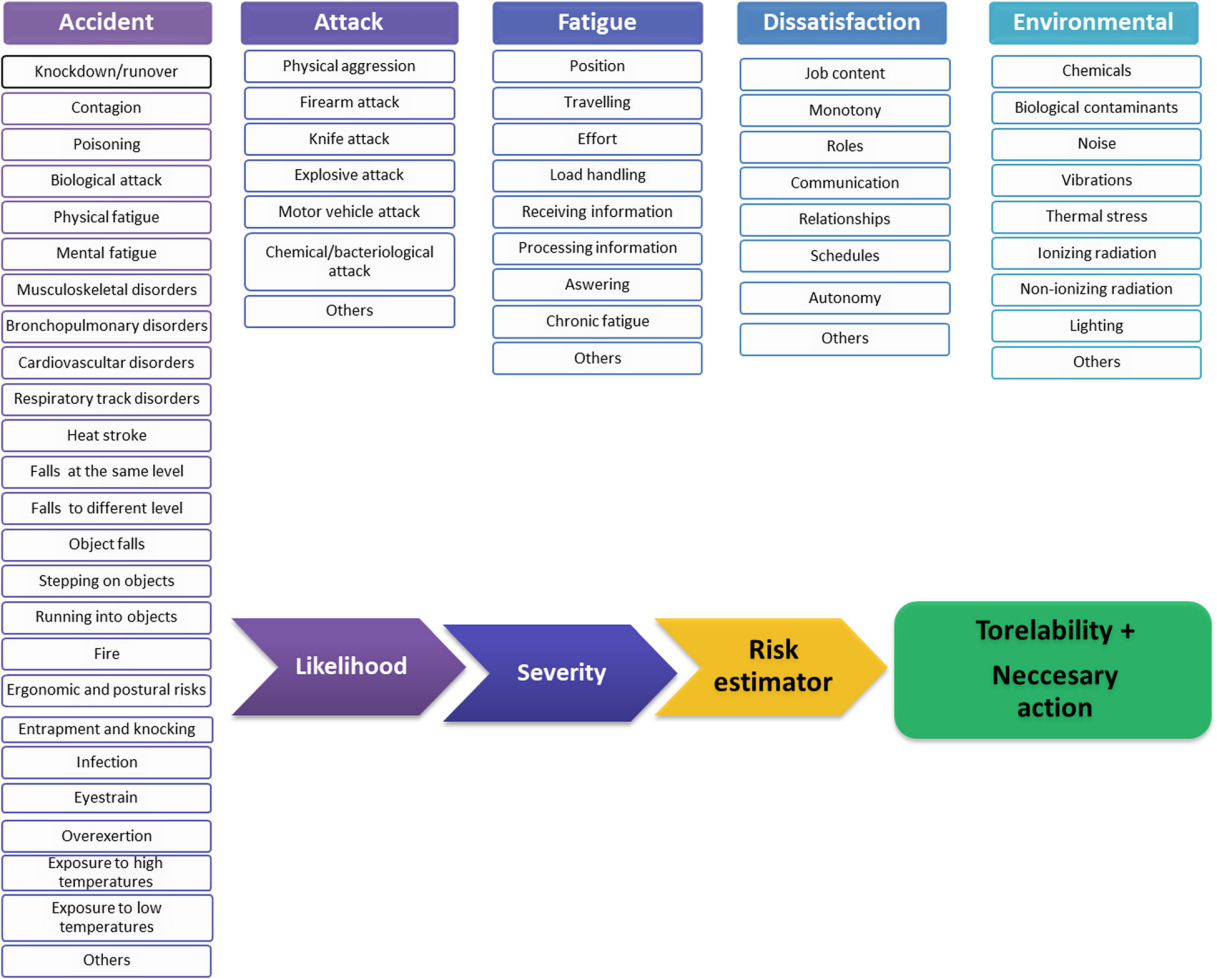
Risk management system module

Once all information has been collected, the preventionist must gather that pertaining to the particular risk and assess its level. The app also allows storage of a photo or video of the risk to be evaluated. Figure 6 depicts all risks included in the app. As can be seen, five general risks (Accident, Attack, Fatigue, Dissatisfaction and Environmental) and their associated actions were considered.

After selecting a specific risk, the user is asked to input its frequency or probability (low, medium or high).

Once all fields have been completed, the app classifies the severity of the risk as slightly harmful, harmful or extremely harmful, according to the INSHT labor risk assessment guide [43]. The last is accompanied by the warning message “WARNING! SERIOUS IMPENDING RISK.” Risk assessments by the app are intended to cater for the needs of public and private security professionals, with special emphasis on ORP requirements. Specifically, the app is designed for police officers working outside the office, where risks are highly diverse, difficult to assess and often unavoidable. For this reason, risk assessment is intended to be a starting point for devising preventive measures, training strategies and equipment handling procedures in order to reduce risk levels for public and private security professionals.

### Evaluation of the app

Satisfaction with the developed app and room for improvement were evaluated through a 10-item questionnaire that was administered to expert users after handling the app. The obtained results are presented in Table 1. As can be observed, the app was assessed for efficiency through the completion rate of tasks and the frequency of incidents and errors in data input. Table 1 shows feedback from the participants in each cycle. As can be seen, all users said that they would use the app frequently; also, they deemed it easy to use (mean complexity score 1.5 out of 5). As can be seen the facility score, Q3, increased from the first cycle (3.6) to the third (4.3), and so did the integration score, Q4, (from 3.4 to 4.3). The overall score for the app increased from 65.9 to 82.1 after improvements.

## Discussion

The proposed app, OTP-PRL, was designed and developed by using a user-centered methodology to ensure that it would meet the actual needs of police officers. One of the greatest advantages of using a backend —and the main reason for using one here— is that it allows apps to be kept alive and in continuous growth. In fact, some errors pinpointed and suggestions made by workgroup members and participants in the laboratory tests were easily corrected or incorporated on the fly.

The iterative methodology of the user-centered design allowed advancing, improving and ensuring that risks in the police domain were duly covered. Also, this methodology, and the results of the usability tests, allowed user satisfaction to be checked. In addition, contributions from experts and users led to easier handling of the app, and also to the incorporation of communication channels (chat and email) for users’ proposals and questions. The app was tested by professionals (law enforcement agents) in both a controlled laboratory environment and a real-life environment (viz., the municipal police headquarters in Cadiz, Spain). The laboratory tests led us to include the attack risk, which is absent from all commercially available apps. One other proposal was to include a specific module for quantifying risk factors (e.g., noise, vibrations, posture) through links to third-party apps.

The app was assessed for efficiency through the consultations by surveys with police officers to receive their complaints and suggestions in order to increase the effectiveness for accomplishing health-related behavior changes in their performance, for example, achieving changes in the way of acting aimed at avoid risks in their performance.

By this way, the app was refined iteratively over three stages (i.e. versions) on the basis of the impression of the police evaluation results (Table 1), each stage leading to a new, improved version. The three refining stages allowed obtaining an improvement in functionality after being evaluated by the police, qualifying the latest version as user-friendly and highly effective for the objectives, meeting all projected design and user requirements.

The final version of this application incorporates the following innovations:

1. It provides accurate information on general and specific regulations on occupational safety and health risk prevention as well as on law enforcing authorities.
2. It allows to produce reports on specific policing Occupational Risks.
3. It allows police officers to make suggestions (preventive measures) after specific interventions.
4. It is fitted with a computer risk assessment feature that each law enforcement officer can customize according to his/her own requirements to perform his/her duties.
5. It provides instant access to an expert consulting chat room.
6. It provides a training platform for both, IOS and Android, as well as a website, all of which will be dedicated to the prevention of Occupational Risks within a policing environment.

There is currently scarce regulation on Occupational Risk Prevention at police environments. Part of it is only related to clerical work, but there is hardly anything related to police patrolling environments, like the ones this App intends to cover. For this reason, the biggest limitation that this app has is due to this lack of legislation in force. In addition, the app so far is only available in two languages English and Spanish. A video demo of the Spanish version is included as Additional file 1.

## Conclusions

A new smartphone app named OTP-PRL was designed and developed by using a user-centered methodology to ensure that it would meet the ORP requirements of police officers. The app provides officers and preventionists with a tool that (*a*) gives accurate information on general and specific legislation on ORP and law enforcement authorities; (*b*) reports on the occupational hazards of policing; (*c*) recommends preventive measures for specific policing activities; (*d*) facilitates computerized risk assessment; (*e*) allows officers to consult experts in relevant areas in real time by chat; and (*f*) offers a training platform for dealing with prevention.

Using a backend keeps the app alive and in continuous growth. A need for an additional module for postural analysis using imaging devices or sensors was noted in the last iteration. Its development, however, was deferred to a subsequent stage as it would require a thorough efficiency, reliability and viability study.

Users evaluated the app through an SUS questionnaire. The final SUS score was 82.3. A field test revealed an increased awareness of police-related occupational risks after using the app in many users.

Based on the results, the proposed smartphone app fulfils its intended purpose so it can be a very useful tool that covers the needs of ORP requirements of police officers.

## Availability and requirements

**Project name:** OTP-PRL

**Project home page:** https://panel.operativatacticapolicial.org/mi_cuenta/instancias

**Operating systems:** iOS and Android.

**Programming languages:** Java (Android) and Objective-c (iOS).

**Other requirements:** Android 4.0 or higher and iOS 11 or higher.

**License:** Səfe Creative – Spain’s Intellectual Property Register (JOSE CARLOS VERA JIMENEZ – File no. 1612280204543).

**Any restrictions to use by nonacademics:** Authorization is required for access to some tasks.

## Supplementary information

**Additional file 1.** Demonstration video of the app (in Spanish).

## Data Availability

All data produced or analyzed during the study are included in this article and its supplementary information files. The app used in the study is available at https://play.google.com/store/apps/details?id=org.operativatacticapolicial.prl Additional information about the app can be found at https://operativatacticapolicial.org/app-otp-prl A demo video for the app is included as supplementary information.

## Abbreviations

ANOVA: Analysis of variance
Apps: Applications
EU: European Union
ICTs: Information and communication technologies
OHS: Occupational health and safety
OR: Occupational risks
ORP: Occupational risk prevention
SLM: Sound level meter
SUS: System Usability Scale
SUS.S: System Usability Scale – Score

## References

[1] Hussain M, Al-Haiqi A, Zaidan AA (2018). A security framework for mHealth apps on android platform. Comput Secur.

[2] Celestina M, Hrovat J, Kardous CA (2018). Smartphone-based sound level measurement apps: evaluation of compliance with international sound level meter standards. Appl Acoust.

[3] de Oliveira Júnior AJ, de Souza SRL, da Cruz VF, Vicentin TA, Glavina ASG (2018). Development of an android APP to calculate thermal comfort indexes on animals and people. Comput Electron Agric.

[4] Carper MM (2017). Multimedia field test thinking about exposures? There’s an app for that!. Cogn Behav Pract.

[5] Melzner J, Heinze J, Fritsch T (2014). Mobile health applications in workplace health promotion: an integrated conceptual adoption framework. Procedia Technol.

[6] Zhao J, Freeman B, Li M (2016). Can mobile phone apps influence people’s health behavior change? An evidence review. J Med Internet Res.

[7] Ramey SL, Downing NR, Franke WD, Perkhounkova Y, Alasagheirin MH (2011). Relationships among stress measures, risk factors, and inflammatory biomarkers in law enforcement officers. Biol Res Nurs.

[8] Nast DR, Speer WS, Le Prell CG (2014). Sound level measurements using smartphone “apps”: useful or inaccurate?. Noise Health.

[9] Ibekwe TS, Folorunsho DO, Dahilo EA, Gbujie IO, Nwegbu MM, Nwaorgu OG (2016). Evaluation of mobile smartphones app as a screening tool for environmental noise monitoring. J Occup Environ Hyg.

[10] Kardous CA, Shaw PB (2014). Evaluation of smartphone sound measurement applications. J Acoust Soc Am.

[11] European Commission Employment (1999). Social Affairs and Equal Opportunities.

[12] Lay AM, Saunders R, Lifshen M (2017). The relationship between occupational health and safety vulnerability and workplace injury. Saf Sci.

[13] Konijn AM, Lay AM, Boot CRL, Smith PM (2018). The effect of active and passive occupational health and safety (OHS) training on OHS awareness and empowerment to participate in injury prevention among workers in Ontario and British Columbia (Canada). Saf Sci.

[14] Estadística de Accidentes de Trabajo del año 2017. Ministerio de Trabajo, Migraciones y Seguridad Social. http://www.empleo.gob.es/es/estadisticas/monograficas_anuales/EAT/2017/index.htm. Accessed 19 July 2018.

[15] Health and safety at work – Eurostat. http://ec.europa.eu/eurostat/web/health/health-safety-work. Accessed 19 July 2018.

[16] Tiesman HM, Gwilliam M, Konda S, Rojek J, Marsh S (2018). Nonfatal injuries to law enforcement officers: a rise in assaults. Am J Prev Med.

[17] Australian Institute of Criminology. Occupational health and safety risks faced by police officers. https://aic.gov.au/publications/tandi/tandi196. Accessed 19 July 2018.

[18] Fowler KA, Betz CJ, Baumgardner JL (2016). Occupational homicides of law enforcement officers, 2003–2013: data from the National Violent Death Reporting System. Am J Prev Med.

[19] Rhee HY, Cho JH, Seok JM (2015). Prevalence of musculoskeletal disorders among Korean police personnel. Arch Environ Occup Health.

[20] Tiesman HM, Swedler DI, Srinivas K, Pollack KM (2013). Fatal occupational injuries among U.S. law enforcement officers: A comparison of national surveillance systems. Am J Ind Med.

[21] Arter ML (2007). Stress and deviance in policing. Deviant Behav.

[22] Ma CC, Andrew ME, Fekedulegn D (2015). Shift work and occupational stress in police officers. Saf Health Work.

[23] O’Hara AF, Violanti JM (2009). Police suicide – a web surveillance of national data. Int J Emerg Ment Health.

[24] Lobel M, Dunkel-Schetter C (1990). Conceptualizing stress to study effects on health: environmental, perceptual, and emotional components. Anxiety Res.

[25] Win KN, Balalla NBP, Lwin MZ, Lai A (2015). Noise-induced hearing loss in the police force. Saf Health Work.

[26] Tan C, Wang Y, Lin M (2018). Long-term high air pollution exposure induced metabolic adaptations in traffic policemen. Environ Toxicol Pharmacol.

[27] Bajaj N, Sharma T, Suneja D, Jain S, Kumar P (2017). Determinants of respiratory and cardiovascular health effects in traffic policemen: a perception-based comparative analysis. J Transp Heal.

[28] Junhua W, Boya L, Lanfang Z, Ragland DR (2016). Modeling secondary accidents identified by traffic shock waves. Accid Anal Prev.

[29] Yan Y, Wu J, Wang X, Sun J, Yuan C, Cheng Y (2010). Investigation on occupational hazards of ultraviolet light, sunscreen awareness and behaviors in Wuhan city traffic police. Zhonghua Lao Dong Wei Sheng Zhi Ye Bing Za Zhi.

[30] Garbarino S (2014). 24-hour work: the interaction of stress and changes in the sleep–wake cycle in the police force. G Ital Med Lav Ergon.

[31] Franklyn-Miller A, Wilson C, Bilzon J, McCrory P (2010). Foot orthoses in the prevention of injury in initial military training: a randomized controlled trial. Am J Sports Med.

[32] Mrena R, Ylikoski J, Kiukaanniemi H, Mäkitie AA, Savolainen S (2008). The effect of improved hearing protection regulations in the prevention of military noise-induced hearing loss. Acta Otolaryngol.

[33] Tsiga E, Panagopoulou E, Niakas D (2015). Health promotion across occupational groups: one size does not fit all. Occup Med (Chic Ill).

[34] Ganesh KS, Naresh AGV, Bammigatti C (2014). Prevalence and risk factors of hypertension among male police personnel in urban Puducherry, India. Kathmandu Univ Med J (KUMJ).

[35] Aleksandra Basinska B, Wiciak I, Maria DA (2014). Fatigue and burnout in police officers: the mediating role of emotions. Polic An Int J Police Strateg Manag.

[36] Instituto Nacional de Seguridad e Higiene en el Trabajo (INSHT). APP – Aplicaciones informáticas para Smartphone o Tablet. http://www.insht.es/portal/site/Insht/menuitem.1f1a3bc79ab34c578c2e8884060961ca/?vgnextoid=3c91f26033bae410VgnVCM1000008130110aRCRD&vgnextchannel=25d44a7f8a651110VgnVCM100000dc0ca8c0RCRD. Accessed 19 July 2018.

[37] Sanner TA, Roland LK, Braa K (2012). From pilot to scale: towards an mHealth typology for low-resource contexts. Heal Policy Technol.

[38] Cai RA, Beste D, Chaplin H (2017). Developing and evaluating JIApp: acceptability and usability of a smartphone app system to improve self-management in young people with juvenile idiopathic arthritis. JMIR mHealth uHealth.

[39] Lerdorf R, Bakken S, Schmid E (1997). PHP Manual, PHP Documentation Group.

[40] Lerdorf R, Tatroe K, MacIntyre P. Programming PHP. 3rd Ed. O'Reilly; 2013. http://web-algarve.com/books/MySQL%20&%20PHP/Programming%20PHP,%203rd%20Edition.pdf. (ISBN: 9781449392272).

[41] Brooke J (2013). SUS: a retrospective. J Usability Stud.

[42] Ley Orgánica 3/2018, de 5 de diciembre, de Protección de Datos Personales y garantía de los derechos digitales (Spanish Organic Law 3/2018, of December 5, on the Protection of Personal Data and guarantee of digital rights).

[43] INSHT. Evaluación de Riesgos Laborales. Ministerio de Trabajo y Asuntos Sociales. 1997. http://www.insht.es/InshtWeb/Contenidos/Documentacion/TextosOnline/Guias_Ev_Riesgos/Ficheros/Evaluacion_riesgos.pdf. Accessed 8 Feb 2019.

